# Dynamics, association, and temporal sequence of cognitive function and frailty: a longitudinal study among Chinese community-dwelling older adults

**DOI:** 10.1186/s12877-023-04328-9

**Published:** 2023-10-13

**Authors:** Kai Cui, Weihan Meng, Zhiqiang Li, Xinning Zeng, Xiaozhe Li, Xiaoyan Ge

**Affiliations:** School of Public Health, Jinzhou Medical University, 40 Songpo Road, Jinzhou, 121000 P. R. China

**Keywords:** Cognition, Frailty, Temporal sequence, Cognitive domain

## Abstract

**Background:**

Little is known about the association of longitudinal dynamics between cognitive function and frailty in Chinese older adults. The temporal sequences between cognitive function and frailty remains unclear. Our study investigates this directionality association using longitudinal data.

**Methods:**

Latent growth and multivariate latent growth models were employed to examine dynamics of cognition and frailty and their association among 2824 older adults in China. Cross-lagged panel analyses were used to assess the temporal sequences between frailty and cognition. The relation between cognitive domains and frailty was also examined using aforementioned methods.

**Results:**

Cognitive function was negatively associated with frailty status. Higher initial level of cognition indicated lower baseline level (*β*=-0.175, *P* < 0.001) and change rate (*β*=-0.041, *P* = 0.002) of frailty. We observed a reciprocal association between frailty and cognitive function rather than a unidirectional causal relationship. The initial cognitive performance for all components were negatively associated with baseline (*β* ranged between − 0.098 to -0.023) and change rate (*β* ranged between − 0.007 to -0.024) of frail status. No consistent associations between change rate of cognitive components and either initial level or change rate of frailty were detected.

**Conclusions:**

Our study detected a reciprocal association between cognition and frailty rather than a unidirectional causal relationship. Our results also revealed different connections between cognitive performance and frailty across diverse cognitive domains.

**Supplementary Information:**

The online version contains supplementary material available at 10.1186/s12877-023-04328-9.

## Background

The development of effective medications and socio-economic conditions improve the health indicators of older populations [1]. However, with the acceleration of ageing and extended lifespans, the suffering of older people from geriatric diseases may be prolonged, increasing the pressure on health-care systems worldwide [1, 2]. China has the fifth largest number of older people worldwide [3]. The increasing burden of ageing populations creates health-care challenges in China [1, 3].

Frailty is a geriatric syndrome, which is characterized by decreased physiological functions, reduced physiological reserve, and increased susceptibility to endogenous or exogenous shock [4, 5]. Previous studies considered frailty a potentially reversible and dynamic entity, and the level of frailty can change bidirectionally over time [2]. Frailty significantly impairs the functional independency of older adults [3] and is associated with unmet care needs, falls and fractures, disability, hospitalizations, lowered quality of life, and mortality [6, 7]. The older population in China over 65 years old showed an 8% frailty prevalence [8].

Cognitive function is the basis of an individual’s capacity to implement appropriate strategies for optimal living [9]. Cognitive impairment is another common geriatric syndrome and often coexists with frailty. This coexistence leads to a vicious cycle in which physical and cognitive decline is further accelerated [10]. Recently, an international consensus group has recognized “cognitive frailty” as the clinical symptom of simultaneous presence of both physical frailty and cognitive impairment in the absence of dementia [11].

Evidence has been accumulating to link frailty with worse global cognitive function [9, 12]. Moreover, associations between frailty and increased risk of future cognitive impairment, incident dementia, and mild cognitive impairment (MCI) have also been reported [7, 13]. Studies showed that the exacerbation of frailty was associated with the rate of cognitive decline among older persons, with a corresponding link between the rates of frailty and cognition changes [14–16]. Besides, subjects with cognitive impairment were independently associated with increased risk of frailty [17]. Recent studies show that frailty and cognitive impairment share common etiologies [1], including oxidative stress, genetic alternations, immune dysfunction, and neuroinflammation [10]. Studies also detected the connection between frailty and certain specific cognitive domains. However, it is unknown which domain is linked to frailty [18, 19].

Convincing evidence from longitudinal analyses of the temporal or causal sequences between frailty and cognition is still lacking [20]. Additionally, most studies on frailty and cognition have been conducted in developed countries; however, little is known about the relationship between frailty and cognition in Chinese community-dwelling older adults [21]. Therefore, the present study investigated the relationship between frailty and global cognitive function and the association between their dynamic changes over time in older Chinese community-dwelling adults. The temporal sequences between frailty and cognition were assessed using a cross-lagged panel design. We also examined the association between domain-specific cognitive performance and frailty.

## Methods

### Data and study participants

The participants were enrolled from the China Health and Retirement Longitudinal Study (CHARLS), which was conducted among Chinese community-dwelling residents aged ≥ 45 years [22, 23]. The baseline survey was conducted in wave 2011 and covered 450 villages and urban communities in China. The participants were resurveyed through face-to-face interviews in waves 2013, 2015, and 2018 [24, 25]. Due to the lack of sufficient information on frailty in wave 2018 [26], this study enrolled participants from waves 2011 to 2015.

**Fig. 1:**
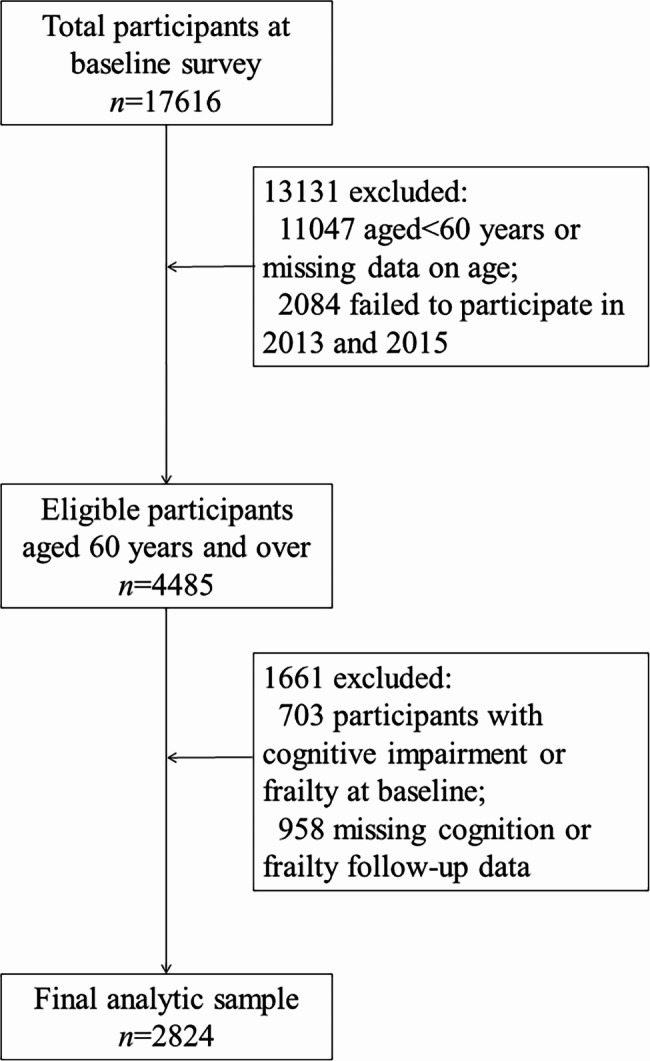
Flow chart of analytic sample

A total of 17,616 community-dwelling adults aged ≥ 45 years participated in CHARLS baseline survey, followed by 18,484 in wave 2013 and 20,991 in wave 2015. In the present study, individuals were eligible if they fulfilled the following criteria: (a) age ≥ 60 years at baseline, (b) without cognitive impairment or frailty at baseline, (c) participated in all three waves from 2011 to 2015, and (d) missed at most one cognitive function measurement or frailty measurement among the three waves. This study included a total of 2,824 older adults; among these, 2,037 had complete data with no missing cognitive or frailty measurements at all three time points. Figure 1 shows the selection process of the analytical sample.

### Global cognitive function measurement

The Telephone Interview of Cognitive Status (TICS-10) was adopted to examine global cognitive function [27–29]. The TICS-10 included the components of time orientation (naming the month, day, year, week, and season), working memory (sequential subtraction of 7 from 100 five times), visual and spatial abilities (redrawing two overlapping pentagons), immediate recall test of memory, and delayed recall test of memory. The participants were asked to recall as many words as they could immediately after hearing a list of ten Chinese nouns. The number of correct words was defined as the immediate recall scores. Several minutes later, the participants were asked to recall the words again; this was considered delayed recall. The scores for global cognitive function were the sum of the correct answers or words and ranged from 0 to 31, with a higher score indicating better global cognition. To eliminate the influence of cognitive impairment at baseline, the participants were grouped every five years of age, and individuals with cognitive scores less than mean-standard deviation (SD) in each age group were excluded [27, 30].

### Frailty measurement

Frailty was measured using Fried’s Physical Frailty Phenotype (PFP), as in previous studies in the CHARLS cohort [31]. Five criteria were used to define frailty: slowness, weakness, exhaustion, inactivity, and weight loss. The participants were asked to walk over a 2.5 m course twice, with slowness defined as an average speed below or equal to the 20th percentile after adjusting for sex and height [31]. A handheld dynamometer was used to assess the handgrip strength twice for each hand, with weakness defined as a maximum of four readings below or equal to the 20th percentile after adjusting for sex and body mass index (BMI). Participants responding ‘a moderate amount of time; 3 to 4 days’ or ‘most of the time; 5 to 7days’ when asked ‘How often during the last week did you feel this way’ to two statements in the Center for Epidemiological Studies-Depression (CESD) scale: ‘I could not get going’ and ‘I felt everything I did was an effort’ met the exhaustion criterion. Participants who walked continuously for < 10 min during a usual week met the criterion for inactivity. Participants who self-reported over 5kg weight loss in the past year or with a current BMI ≤ 18.5kg/m^2^ met the criterion for weight loss. The number of criteria met was used to assess frailty status. Individuals who met no criteria were considered ‘non-frail’ or ‘robust’; those who met 1–2 criteria were deemed ‘prefrail’; and those who met ≥ 3 criteria were defined as ‘frail’. Individuals who missed two or more of the five frailty criteria were excluded.

### Covariates

The potential covariates included baseline measurements of age, sex, residence region, education level, marital status, status of current smoking and drinking, number of comorbidities, and depression in wave 2011. As mentioned in previous studies, the comorbidities included hypertension, diabetes mellitus, dyslipidemia, heart disease, stroke, cancer, lung disease, arthritis, kidney disease, digestive disease, and asthma [28, 32]. Depression was measured using 10 items of the CESD scale. The scores ranged from 0 to 30, with greater values representing higher levels of depressive symptoms [33].

### Statistics analyses

Mean with SD and frequencies with percentages were calculated as descriptive statistics for continuous and categorical variables, respectively. Comparisons among the three waves were performed using repeated-measures analysis of variance and the Friedman’s test. Linear regression was employed to assess the impact of frailty on cognition at each cross-sectional time point, after adjusting for the covariates. Additionally, ordinal regression was utilized to investigate the influence of cognition on frailty.

The latent growth model was used to describe the course of cognition and frailty across the three waves after adjusting for the effects of baseline covariates. Longitudinal changes were examined using intercepts and slopes as latent variables. The intercept represented the average level at baseline, while the slope represented the average rate of change per unit time over the follow-up period. Additionally, the variances of the intercept and slope were estimated to indicate individual differences. The parameters were estimated using maximum likelihood estimation. We used a multivariate latent growth model to evaluate the relationship of longitudinal dynamics between cognition and frailty [34]. The unstandardized pathway coefficients between the parameters were also estimated.

The temporal sequences between cognition and frailty were examined using a cross-lagged model after adjusting for the effect of baseline covariates without restricting the cross-lagged effects across waves to be equal. The standardized autoregressive and cross-lagged pathway coefficients were also estimated. The differences between the two cross-lagged pathway coefficients in each time span were tested using Fisher’s Z-test [35].

Sensitivity analyses were performed using the subset of complete data. The main analyses of the multivariate growth and cross-lagged models were repeated to validate the robustness of the results. Additionally, the dynamics and associations between cognitive domains, with the components of TICS-10 as the indicators, and frailty were explored using the aforementioned methods.

The comparative fit index (CFI), standardized root mean square residual (SRMR), and root mean square residual (RMR) were used to evaluate the goodness of fit of each model. Indices for CFI ≥ 0.90 and SRMR and RMR ≤ 0.08 indicated an acceptable fit. The full information maximum likelihood (FIML) method was used to handle missing data.

All analyses were conducted in R software version 4.2.2 (The R Foundation for Statistical Computing, Vienna, Austria, https://www.r-project.org). All tests were two-tailed and statistical significance was set at *P* < 0.05.

## Results

### Demographic characteristics and cross-sectional associations between cognitive function and frailty

Baseline demographic characteristics of the participants are shown in Table 1. The mean age of participants was 67.069 ± 5.867 years and 55.17% were male. The majority were rural residents, with a relatively low education level, never smoker, and non-drinkers. The scores of global cognitive function and frailty status in the three waves are shown in Table 2. The results showed that cognitive function decreased over time (*P* < 0.001). The cross-sectional associations are shown in Additional file 1. In every wave of the survey, significant negative associations were identified between cognition and frailty, and the unstandardized regression coefficients (*β*) ranged from − 0.045 to -1.400 (*P* < 0.001).

**Table 1:** Demographic characteristics of baseline (wave 2011)

Variables	Level	Mean ± SD	*N* (%)
Age		67.069 ± 5.867	
Age group	60-		1202 (42.56)
	65-		764 (27.05)
	70-		491 (17.39)
	75-		367 (13.00)
Sex	Male		1558 (55.17)
	Female		1266 (44.83)
Residence region	Rural		2202 (77.97)
	Urban		622 (22.03)
Marital status	Married		548 (19.41)
	Others		2276 (80.59)
Education level	No formal education/illiterate		765 (27.09)
	Can read or write but did not finish elementary school		610 (21.06)
	Elementary school		853 (30.21)
	Middle school		416 (14.73)
	High school or above		180 (6.37)
Smoking status	Current		939 (33.25)
	Former		336 (11.90)
	Never		1549 (54.85)
Drinking status	Drinker		759 (26.88)
	Non-drinker		2065 (73.12)
Number of comorbidities	0		785 (27.80)
	1		858 (30.38)
	2		599 (21.21)
	≥ 3		582 (20.61)
Depression		7.989 ± 5.970	

**Table 2:** Comparison of frailty and global cognitive function among three waves

Variables	Wave 2011	Wave 2013	Wave 2015	* F/χ* ^*2*^	*P*
Frailty					
Robust/non-frail	1035 (36.65)	903 (31.98)	811 (28.72)	56.351 ^a^	< 0.001
Prefrail	1789 (63.35)	1437 (50.89)	1167 (41.32)		
Frail		265 (9.38)	212 (7.51)		
Missing		219 (7.75)	634 (22.45)		
Global cognition	14.025 ± 4.756	13.096 ± 5.938	12.104 ± 5.910	199.850	< 0.001

^a^: Friedman test for related samples rank-sum test with missing data omitted.

### Dynamics of cognitive function and frailty

For cognition, the latent growth model showed an acceptable fit for the data (CFI = 1.000, SRMR = 0.002, RMR = 0.013). The initial average TICS-10 score was 11.512 (*P* < 0.001) and decreased with a rate of 0.641 (*P* < 0.001) at each time point during the follow-up period. Additionally, the intercept and slope were not significantly correlated (*β*=-0.200, *P =* 0.296). For frailty, the model fit to the data well (CFI = 0.988, SRMR = 0.005, and RMR = 0.002). The mean intercept and slope were 0.698 (*P* < 0.001) and − 0.054 (*P* = 0.041), respectively. We observed no significant association between the intercept and slope (*β* = 0.004, *P* = 0.211).

### Associations of dynamics between cognitive function and frailty

**Fig. 2:**
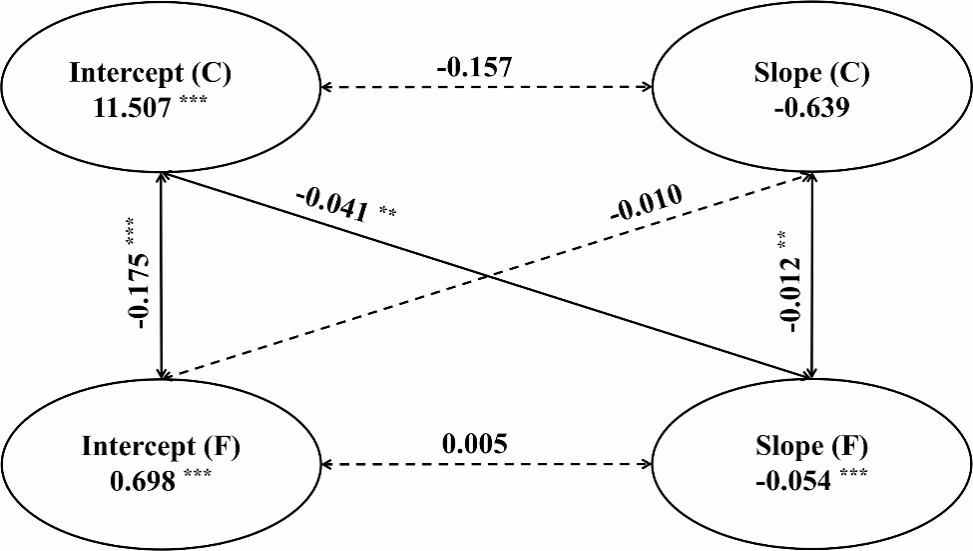
Associations of dynamics between cognitive function and frailty. The models were adjusted for baseline covariates, including age, sex, residence region, marital status, education level, current smoking and drinking status, number of comorbidities, and depression symptoms. For brevity, the covariates are omitted in this figure. The dashed lines indicate the non-significant path coefficients. C, cognitive function; F, frailty; ^**^P < 0.01; ^***^P < 0.001

The indices indicated a good fit between the model and the data (CFI = 0.997, SRMR = 0.006, and RMR = 0.028). As shown in Fig. 2, no significant relationship was observed between the intercept and slope for frailty or cognition (*P* = 0.405 and *P* = 0.139, respectively). The intercept of cognition was negatively associated with the intercept of frailty (*P* < 0.001), and the slope of frailty (*P* = 0.002). Additionally, we observed a negative association between the slope of cognition and that of frailty (*P* = 0.007), suggesting that a higher rate of change in cognition was associated with a lower rate of frailty. The intercept of frailty was not associated with the slope of cognition (*P* = 0.355), indicating that initial frailty status did not influence changes in cognition.

### Temporal sequence between cognitive function and frailty

**Fig. 3:**
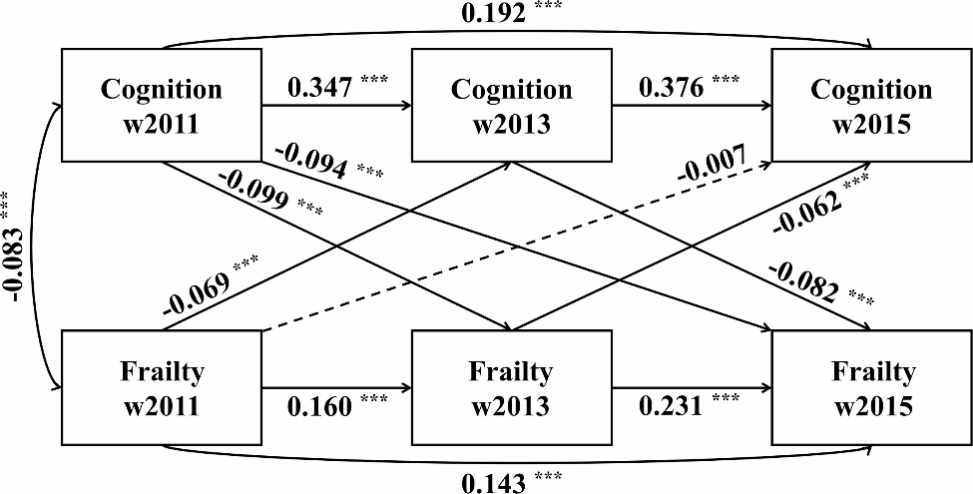
Temporal sequence between cognitive function and frailty. The models were controlled for baseline covariates. Dashed lines, non-significant path coefficients. ^***^P < 0.001

Figure 3 shows the cross-lagged model estimates of the reciprocal association between cognition and frailty. The results suggested an acceptable fit for the data (CFI = 0.991, SRMR = 0.010, and RMR = 0.041). Significant associations were detected in the autoregressive pathways for both cognition and frailty. That is, initial cognition predicted future cognition, and baseline frailty predicted future frailty status. We observed the significant negative cross-lagged effect between cognition and frailty. This indicated that lower levels of cognition subsequently predicted higher frailty scores and vice versa. The standardized path coefficient from cognition in 2011 to frailty in 2013 was greater than that from frailty in 2011 to cognition in 2013 (-0.099 vs. -0.069, respectively); however, the difference between these two coefficients was not significant (*P* = 0.256). Similarly, the coefficient from cognition in Wave 2013 to frailty in Wave 2015 was greater than that from frailty to cognition (-0.082 vs. -0.062); however, the difference was not statistically significant (*P* = 0.450). This indicated a reciprocal association between cognition and frailty, rather than a unidirectional causal relationship.

### Sensitivity analyses

The results suggested an acceptable fit for the data in the sensitivity analyses (multivariate growth model: CFI = 0.997, SRMR = 0.006, and RMR = 0.024; cross-lagged model: CFI = 0.989, SRMR = 0.010, and RMR = 0.039). As shown in Additional files 2 and 3, the results are similar to those of the main analyses, suggesting the robustness of the present study.

### Associations between cognitive components and frailty

We conducted multivariate growth and cross-lagged model analyses for the dynamics of frailty and the five cognitive components of the TICS-10. The fit indices, shown in Additional file 4, indicated that the models were acceptable for the data.

**Fig. 4:**
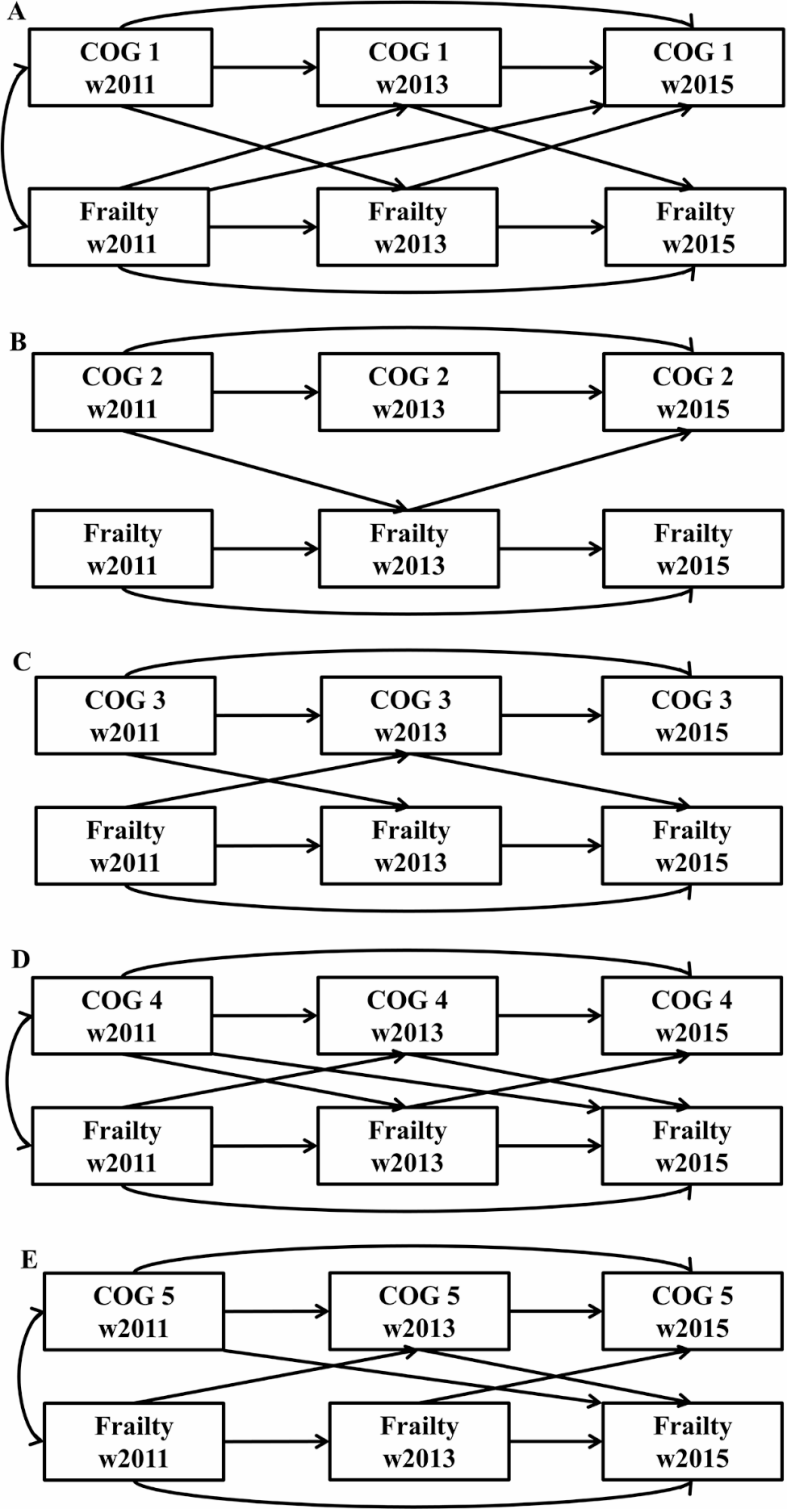
Associations between cognitive components and frailty. The models were controlled for baseline covariates. The solid lines indicate that the path coefficient is statistically significant (*P* < 0.05). (**A**) COG 1 for the cognitive component of time orientation; (**B**) COG 2 for the cognitive component of working memory; (**C**) COG 3 for the cognitive component of visual and spatial abilities; (**D**) COG 4 for the cognitive component of the immediate recall test of memory; (**E**) COG 5 for the cognitive component of the delayed recall test of memory

Table 3 lists the parameters of the multivariate growth model. The slope for each cognitive component decreased over time. The intercept of each cognitive component was negatively associated with both the intercept and slope of frailty, indicating that higher initial levels for different cognitive domains were associated with lower baseline and change rate of frailty. Moreover, no consistent relationship between the slope of cognitive performance and either the frailty intercept or frailty slope was identified across the components. Figure 4 shows the results of the cross-lagged model. Different cross-lagged pathways were observed in five cognitive components. No significant difference was detected between the cross-lagged coefficients. The reciprocal associations appeared to differ between diverse cognitive domains and frailty status.

**Table 3:** Associations between cognitive components with frailty

Parameters	Cognitive components ^a^				
	1	2	3	4	5
Intercept (C)	**3.846** ^***^	**3.172** ^***^	**0.655** ^***^	**3.916** ^***^	**2.976** ^***^
Slope (C)	**-0.097** ^***^	**-0.062** ^***^	**-0.022** ^***^	**-0.139** ^***^	**-0.162** ^**^
Intercept (F)	**0.649** ^***^	**0.649** ^***^	**0.649** ^***^	**0.649** ^***^	**0.649** ^***^
Slope (F)	**0.030** ^***^	**0.029** ^***^	**0.029** ^***^	**0.029** ^***^	**0.029** ^***^
**Pathway coefficients**					
Intercept (C)-slope (C)	-0.003	0.005	-0.003	0.040	-0.012
Intercept (F)-slope (F)	0.006	0.005	0.005	0.006	0.006
Intercept (C)- Intercept (F)	**-0.090** ^***^	**-0.071** ^***^	**-0.023** ^***^	**-0.098** ^***^	**-0.093** ^***^
Slope (C)- Intercept (F)	**-0.007** ^*^	0.001	-0.001	-0.006	**-0.012** ^*^
Intercept (C)- Slope (F)	**-0.016** ^***^	**-0.024** ^***^	**-0.007** ^***^	**-0.023** ^***^	**-0.022** ^***^
Slope (C)- Slope (F)	**-0.004** ^**^	-0.004	0.000	**-0.003** ^*^	-0.004

^a^: 1 time orientation, 2 working memory, 3 visual and spatial abilities, 4 immediate recall test of memory, 5 delayed recall test of memory; ^*^: *P* < 0.05; ^**^: *P* < 0.01; ^***^: *P* < 0.001; C: cognition; F: Frailty

## Discussion

This study explored the association between cognitive function and frailty in Chinese older adults. We confirmed the reciprocal association between frailty and cognitive function and identified diverse connections between cognition and frailty across different cognitive domains.

In this study, the change rates of both cognition and frailty did not depend on their initial levels; therefore, the change rate may be either fast or slow, even if the initial level is high. Currently, there was a contentious debate regarding the relationship between baseline cognitive function and the subsequent changes. In Ma’s research, older adults with subjective memory decline with higher global cognition showed a less rapid cognitive decline [36]. However, in another study, baseline cognition was positive associated with cognitive decline [37]. Besides, previous studies have demonstrated that the level of frailty can change bidirectionally over time [2]. This, to some extent, explains the absence of an identified correlation between baseline frailty and its change rate in our research. It seems that regardless of the baseline cognition or frailty status of older adults, their subsequent change rates exhibit notable heterogeneity. This suggests that regular monitoring of global cognitive function and frailty status in community-dwelling older adults is crucial for preventing cognitive impairment and adverse health outcomes.

Cognitive impairment and frailty share common biological pathways. For example, oxidative stress contributes to frailty and impaired cognitive performance. Reactive oxygen species (ROS) induce changes at the cellular level and lead to systemic responses that influence frailty [38]. Meanwhile, the brain is particularly susceptible to the harmful effects of oxidative damage owing to its deficiency in free radical protective antioxidant compounds and the non-renewability of nervous tissue [14, 15, 39]. In the present study, older adults with better cognitive function had a lower change rate of frailty as well as initial frailty status. We inferred that higher cognitive function may indicate a lower level of adverse factors, more neural reserve, stronger tolerance, or more stability for degeneration of physiological systems caused by ageing. This strong association further confirms that cognitive impairment and frailty share a common pathological basis.

The temporal sequence of cognitive function and frailty remains controversial owing to limitations in study design and analysis methods [40–43]. Our results indicated a reciprocal relationship between cognitive function and frailty rather than a unidirectional causal association. In Zhao’s study, physical function was assessed through three tests capturing the Chinese older people’s physical performance, including “stand up from sitting in a chair without using hands”, “stand up to pick up a book from floor”, and “turn around 360° without help” [44]. The results demonstrated a positive reciprocal relationship between physical and cognitive functions, and showed no evidence suggesting that the predictive effect of physical performance on subsequent cognition was significantly larger than that of cognition on subsequent physical performance or vice versa [44]. Our findings are consistent with Zhao’s study [44]. Our findings demonstrate that the interventions for preventing against frailty, such as leading a physically active lifestyle, contribute to decelerate cognitive decline; Conversely, interventions aimed at preserving cognitive function or decelerating cognitive decline will also contribute to enhance the physical function of older adults and sustaining their independence in daily activities. Results from randomized controlled trials also demonstrates that physical activities can enhance cognitive function in older adults, and cognitive training improve their physical function [45–47]. Attributed to the common biological pathways and etiologies, cognitive decline and frailty may coexist or occur simultaneously, which create the construct of cognitive frailty [48]. As a complete understanding of the underpinning biological basis of cognitive decline and frailty remains fragmented [49], further longitudinal studies are needed to elucidate the relationship between these disorders.

Cognitive domains are reportedly associated with frailty; however, whether the link differs by cognitive domains remains unclear. Chen et al. reported that all domains measured using Montreal Cognitive Assessment were associated with frailty status, except for orientation, when comparing frail and non-frail individuals [50]. In another recent study in older adults in US communities, frail individuals showed significant declines in cognitive function compared to non-frail adults across all domains except for immediate word recall [51]. In our study, the association between the change rate of cognitive performance with level or change rate of frailty was diverse across cognitive domains. The pathways in the cross-lagged models for every cognitive component also supported the idea that the diverse connections between cognitive performance and frailty across different cognitive domains. Further studies on the underlying biological mechanisms are required.

This study had several limitations. First, the CHARLS lacked neuroimaging data and neuropsychological tests to assess performance in cognitive domains. We used the components of the TICS-10, which correlate well with the Mini-Mental State Examination, as substitute indicators of cognitive domains, which have been validated in other studies [52, 53]. Second, the measurement of global cognitive function and frailty was difficult in older adults. This study excluded individuals who lacked one cognitive function measurement or information on frailty. Selection bias could, to some extent, contribute to the overestimation or underestimation of the association between cognition and frailty. Furthermore, due to the lack of relevant data in CHARLS database, the influence of residual confounding was unable to be eliminated in this study, such as the apolipoprotein E genotype. Finally, the follow-up time of 4 years was relatively short across the life course. Longitudinal studies with longer observation periods are warranted.

## Conclusions

The results of our study illustrated the relationship between the dynamics of cognitive function and frailty among community-dwelling older adults in China. We observed a reciprocal association between cognition and frailty rather than a unidirectional causal relationship. Our results also revealed different connections between cognitive performance and frailty across diverse cognitive domains. Accordingly, regular monitoring of global cognitive function and frailty is crucial for the ageing Chinese population.

### Electronic supplementary material

Below is the link to the electronic supplementary material.

Supplementary Material 1

Supplementary Material 2

Supplementary Material 3

Supplementary Material 4

## Data Availability

The data that support the findings of this study are available in the China Health and Retirement Longitudinal Study (CHARLS) repository, http://charls.pku.edu.cn.

## List of abbreviations

MCI: Mild cognitive impairment
CHARLS: China Health and Retirement Longitudinal Study
TICS: Telephone Interview of Cognitive Status
SD: Standard deviation
PFP: Physical Frailty Phenotype
BMI: Body mass index
CESD: Center for Epidemiological Studies-Depression
CFI: Comparative fit index
SRMR: Standardized root mean square residual
RMR: Root mean square residual
FIML: Full information maximum likelihood
ROS: Reactive oxygen species
