# Supplementary material for: Dynamics, association, and temporal sequence of cognitive function and frailty: a longitudinal study among Chinese community-dwelling older adults
Source: BMC Geriatr. 2023 Oct 13;23:658. doi: 10.1186/s12877-023-04328-9 (PMC10571451; doi:10.1186/s12877-023-04328-9)
Supplement: Supplementary file 1 — Supplementary Material 1 [file 12877_2023_4328_MOESM1_ESM.docx]

**Additional file 1**

Cross-sectional associations between cognition and frailty in every wave after adjusting for the covariates

| **Dependent variable** | **Independent variable** | ***β* (SE) ^a^** | ***P*** | ***R^2^*** |
| --- | --- | --- | --- | --- |
| Cognition (2011) | Frailty (2011) | -0.701 (0.158) | <0.001 | 0.312 |
| Cognition (2013) | Frailty (2013) | -1.051 (0.148) | <0.001 | 0.347 |
| Cognition (2015) | Frailty (2015) | -1.400 (0.161) | <0.001 | 0.365 |
| Frailty (2011) | Cognition (2011) | -0.045 (0.010) | <0.001 | 0.054 |
| Frailty (2013) | Cognition (2013) | -0.061 (0.009) | <0.001 | 0.098 |
| Frailty (2015) | Cognition (2015) | -0.079 (0.010) | <0.001 | 0.119 |

^a^: Adjusted for age group, residence region, education, married status, sex, smoking status, drinking status, and number of comorbidities.
