# Supplementary material for: Dynamics, association, and temporal sequence of cognitive function and frailty: a longitudinal study among Chinese community-dwelling older adults
Source: BMC Geriatr. 2023 Oct 13;23:658. doi: 10.1186/s12877-023-04328-9 (PMC10571451; doi:10.1186/s12877-023-04328-9)
Supplement: Supplementary file 2 — Supplementary Material 2 [file 12877_2023_4328_MOESM2_ESM.docx]

**Additional file 2**


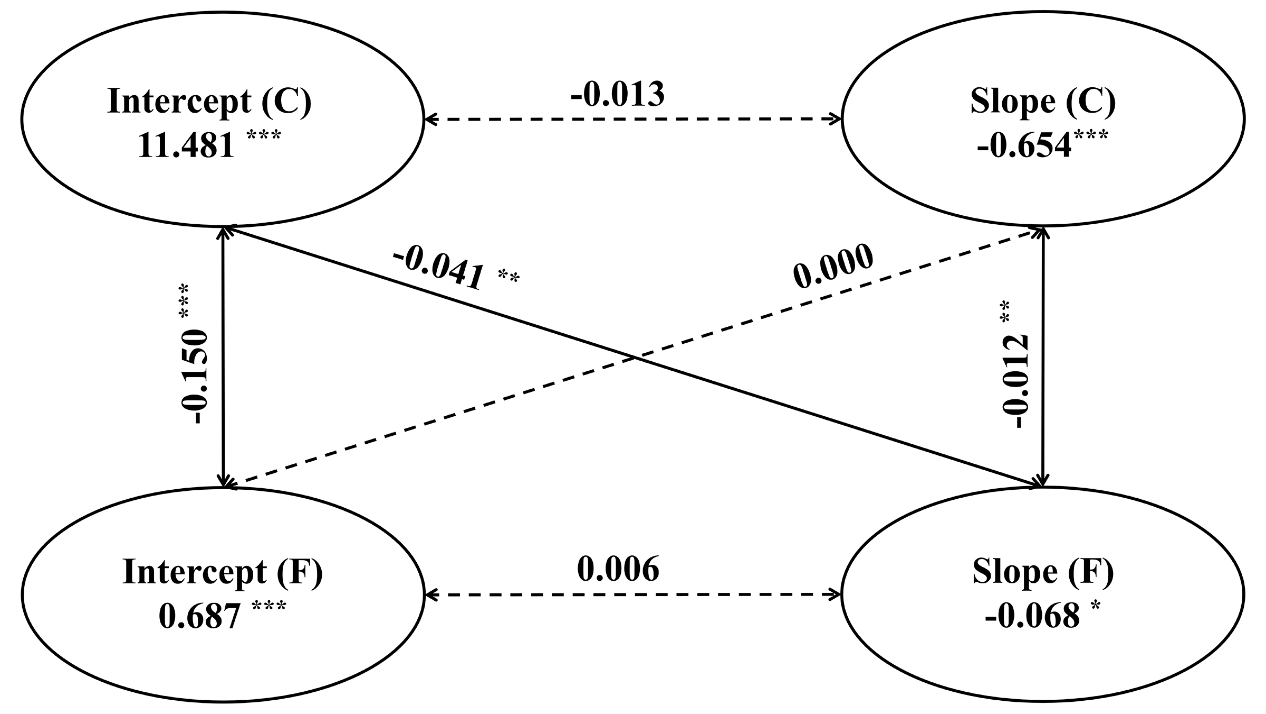


**Associations of dynamics between cognitive function and frailty in sensitivity analyses**

The baseline covariates were adjusted in the models. The dashed lines indicate the non-significant path coefficients. C: Cognitive function; F: Frailty; ^**^: *P*<0.01; ^***^: *P*<0.001.
