# Supplementary material for: Dynamics, association, and temporal sequence of cognitive function and frailty: a longitudinal study among Chinese community-dwelling older adults
Source: BMC Geriatr. 2023 Oct 13;23:658. doi: 10.1186/s12877-023-04328-9 (PMC10571451; doi:10.1186/s12877-023-04328-9)
Supplement: Supplementary file 4 — Supplementary Material 4 [file 12877_2023_4328_MOESM4_ESM.docx]

**Additional file 4**

Fit indices of multivariate growth models and cross-lagged models for cognitive components and frailty

| **Components** | **CFI** | **SRMR** | **RMR** |
| --- | --- | --- | --- |
| **Multivariate growth model** |  |  |  |
| Orientation | 0.974 | 0.026 | 0.024 |
| Working memory | 0.974 | 0.024 | 0.021 |
| Visual and spatial abilities | 0.969 | 0.023 | 0.020 |
| Immediate recall test of memory | 0.978 | 0.021 | 0.012 |
| Delayed recall test of memory | 0.953 | 0.028 | 0.035 |
| **Cross-lagged model** |  |  |  |
| Orientation | 0.994 | 0.008 | 0.008 |
| Working memory | 0.993 | 0.007 | 0.008 |
| Visual and spatial abilities | 0.993 | 0.007 | 0.008 |
| Immediate recall test of memory | 0.995 | 0.006 | 0.002 |
| Delayed recall test of memory | 0.988 | 0.009 | 0.011 |
